# The relationship between self-reported mental health and redeemed prescriptions of antidepressants: a register-based cohort study

**DOI:** 10.1186/s12888-016-0893-7

**Published:** 2016-06-07

**Authors:** Louise Sjørslev Frandsen, Line Bilgrav Villumsen, Cathrine Fonnesbech Hjorth, Berit Jamie Nielsen, Line Rosenkilde Ullits, Christian Torp-Pedersen, Henrik Bøggild, Charlotte Overgaard

**Affiliations:** Public Health and Epidemiology Group, Department of Health Science and Technology, Aalborg University, Niels Jernes Vej 14, DK-9220 Aalborg, Denmark; Department of Clinical Epidemiology, Aalborg University Hospital, Sdr. Skovvej 15, DK-9000 Aalborg, Denmark

**Keywords:** Mental health, Antidepressant agents, Young, Sex differences

## Abstract

**Background:**

Poor mental health is a major problem in most western societies, especially predominant among young adults. However, associations of self-reported poor mental health with subsequent psychiatric or medical treatment are unknown. We examined the relation between self-reported mental health and redeeming prescriptions of antidepressants among three age groups.

**Methods:**

We analyzed data from 16,233 individuals aged 16 years and over randomly selected to participate in the 2010 North Denmark Region Health Survey completed in February 2010. Mental health was defined according to the Short-Form 12 instrument (SF-12) and dichotomized into poor and good. Outcome data were retrieved from administrative information on redeemed prescriptions of antidepressants between February 2010 and December 2012. Crude cumulative incidence curves were produced to illustrate the probability of redeeming new prescriptions of antidepressants over time. Cox regression analysis was used to estimate risk of redeeming prescriptions of antidepressants when having poor mental health, adjusted for preselected explanatory covariates.

**Results:**

Among the young (16–29 years-old), 620 (23 %) participants suffered from poor mental health. Among the adults (30–59 years-old) and elderly (60 years-old or over), 1592 (18 %) participants and 723 (15 %) reported poor mental health, respectively. Overall, women were more likely than men to rate their mental health as poor. For all age groups, there was an increased probability for redeeming prescriptions of antidepressants when having poor mental health. The hazard ratio [HR] for redeeming prescriptions of antidepressants for those reporting poor versus good mental health, adjusted for sex, ethnicity, marital status, education level, occupational status, smoking and physical activity was 3.1 (95 % confidence interval [CI] 2.20–4.29) for young participants. For adults, the HR was 2.3 (95 % CI 1.86–2.78) and for elderly, it was 3.5 (95 % CI 2.66–4.57).

**Conclusion:**

Self-reported poor mental health was more frequent among younger than older participants. Overall, antidepressants were the most often used treatment. An increased probability of redeeming antidepressant prescriptions when having self-reported poor mental health was observed in all age groups. These findings suggest that frequent reporting of poor mental health is a common issue for all age groups that needs more attention.

**Electronic supplementary material:**

The online version of this article (doi:10.1186/s12888-016-0893-7) contains supplementary material, which is available to authorized users.

## Background

Poor mental health is a major problem in most western societies and leads to increased morbidity [1–5]. Poor mental health is ascribed to young adults in particular [1, 2, 5], as also indicated by an increasing rate of antidepressant treatment in this group [6–8].

The consumption of antidepressants has increased markedly in many countries, including Denmark. In the 23 Organisation for Economic Co-operation and Development (OECD) countries, the mean consumption of antidepressants increased with 70 % from 2000 to 2011, as the mean consumption of antidepressants was 33 Defined Daily Dose (DDD) per 1000 people per day in 2000 and 56 DDD per 1000 people per day in 2011 [9]. In Denmark, the mean consumption of antidepressants doubled, from 35 DDD per 1000 people per day in 2000 to 85 DDD per 1000 people per day in 2011, ranking Denmark as number four among OECD countries, only surpassed by Canada (third), Australia (second) and Iceland (highest) [9]. The increased use was particularly marked among young people (both men and women aged between 18 to 24 years) for whom the proportion of redeemed antidepressant prescriptions rose with 148 % from 2001 to 2010, as 23 people per 1000 people redeemed antidepressants in 2001 and 57 people per 1000 people redeemed antidepressants in 2010 in Denmark [7]. Antidepressants are sometimes supplemented with sedatives [10, 11]. When treating people suffering from poor mental health, psychological therapy is also commonly applied [12].

Several surveys have found that poor mental health is most frequently reported by young women [13–15]. In Denmark, an increase from 15.8 % in 2010 to 17.5 % in 2013 has been observed in self-reported poor mental health among young women [16, 17]. Other studies have demonstrated an association between socioeconomic status and mental health as well as the use of antidepressants [18–20]. However, the associations between self-reported poor mental health with subsequent psychiatric or medical treatment, long-term morbidity, and serious health problems are currently unknown.

Previous studies has established sex, age, and socioeconomic status as risk factors for poor mental health and medication use [19–23]. To further examine the association between mental health and conventional medical or psychological treatment, we performed analyses of the relationship between mental health, use of antidepressant medication and sedatives, and use of psychological therapy.

The aim of our study was to examine (i) the proportions of self-reported mental health states and types of conventional medical or psychological treatment and (ii) the relationship between poor mental health and redemption of prescriptions of antidepressants among different age groups. We hypothesized (i) that poor mental health is most frequent among young women, antidepressants are the most used conventional medical or psychological treatment, and (ii) poor mental health increases the risk of requiring conventional medical or psychological treatment.

## Methods

### Study population and data

**Fig. 1:**
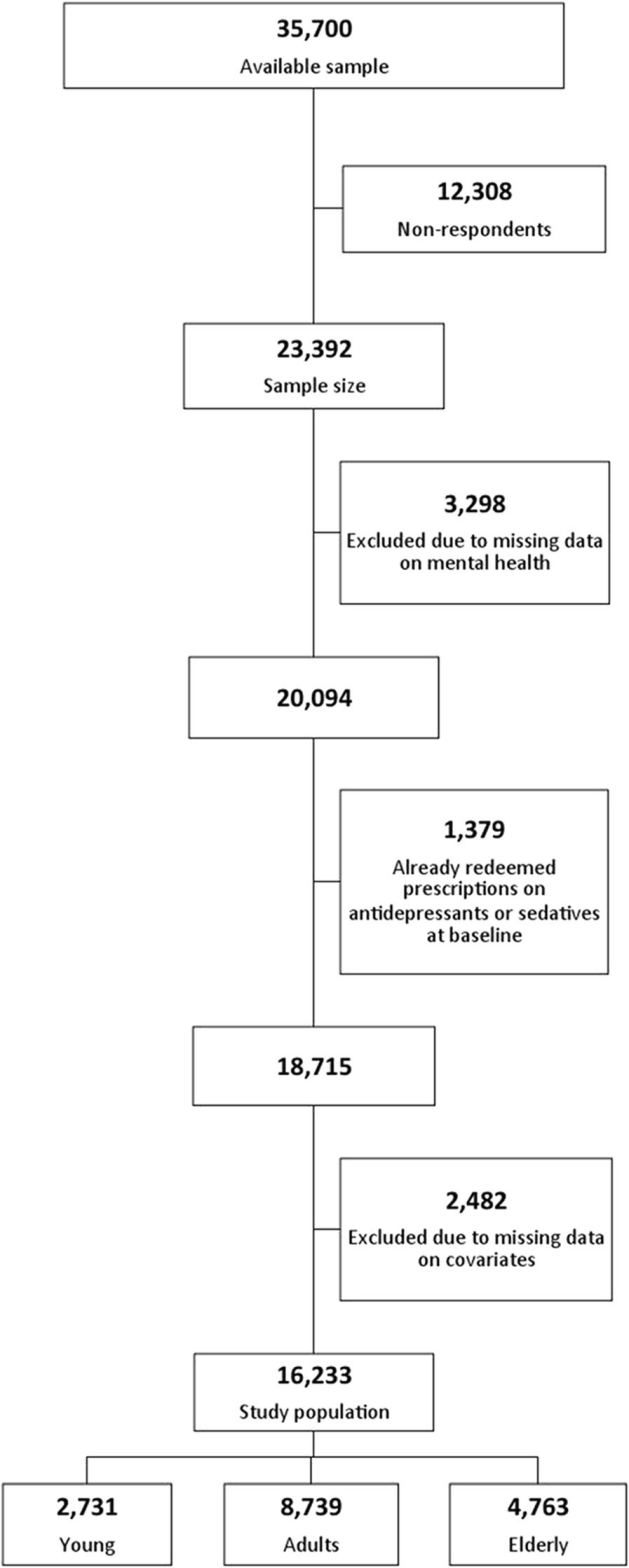
Flowchart of the selection process. Flowchart illustrating the selection process of the study. The three age groups are defined as; young (16–29 years of age), adults (30–59 years of age) and elderly (≥ 60 years of age). From the North Denmark Region Health Survey 2010 [24]

This was a register-based cohort study with a follow-up between 1 February 2010 to 31 December 2012, based on the North Denmark Region Health Survey 2010 [24]. A postal questionnaire containing 76 questions including the Short-Form 12 (SF-12) items, was submitted to 35,700 persons aged 16 years and over [24, 25]. The sample was drawn randomly within 11 municipalities from a population of 469,998 inhabitants in the Civil Registration System [24, 26, 27]. The sampling design gave citizens in different municipalities unequal selection probabilities. Citizens living in a large municipality were less likely to be selected for the survey compared with citizens living in municipalities with a small population. Failing to account for this could lead to biased parameter estimates and therefore analyses were conducted applying a design of weights to adjust for the different selection probability. The data collection process extended between 5 February and 22 March 2010. Reminders were sent during mid-February 2010 and the first part of March 2010 to citizens who had not returned the health survey, and a total of 23,392 participants returned the questionnaire [24]. From the sample, 20,094 participants answered the items on mental health. Regarding the redemption of antidepressant prescriptions, 1298 participants at baseline had already redeemed prescriptions and of those 1189 continued using already-filled prescriptions. Thus, they were excluded from the analysis. For sedatives, 266 participants redeemed prescriptions at baseline, and of those 190 participants continued already-filled prescriptions of sedatives. Thus, they also were excluded. Looking specifically at participants’ utilization of a psychologist no participants were excluded from the study due to data on psychological therapy before 2011 were insufficient recorded. Because of missing data on covariates, 2482 participants were excluded. This resulted in a study population of 16,233 participants that were not being treated with antidepressants or sedatives at baseline and had complete information on all of the evaluated variables. The overall response rate was 45.6 % (44.9 % among men) (Fig. 1).

### Data sources

By use of the unique civil registration number (CPR number) assigned to all Danish citizens, linkage between data from the health survey and administrative registers were made [26, 28]. We obtained data on prescriptions, utilization of healthcare services, demographic information and socioeconomic information from six registers. The Danish National Prescription Registry contains complete data on all claimed prescriptions in the country [29]. The National Health Service Register holds information about consultations reported by every healthcare professional that provides services in the tax-funded public healthcare system in Denmark [30]. The Central Population Register includes data on every person living in Denmark, with dates of birth, death, and sex [28]. The Population’s Education Register provides information on the complete education programs that are authorized by the Danish Ministry of Education [31]. The Danish Income Statistics Register contains information on income taxes for Danish residents [32]. The Danish Register for Evaluation of Marginalization (DREAM) contains information of the public transfer payments done on a weekly basis [33, 34].

### Outcome measure

Prescription data of drugs within ATC-codes (Anatomical Therapeutic Chemical Classification System) [35] N06A (antidepressants), N05B (anxiolytics) and N05C (hypnotics) in the period between 1 August 2009 to 31 December 2012 was drawn from The Danish National Prescription Registry [29]. Information on utilization of a psychologist was defined as the first consultation for psychological therapy in the period from 1 January 2011 to 31 December 2012. This time period was chosen because of insufficient data on psychological therapy before 2011. This information was obtained from The National Health Service Register [30].

### Exposure measure

The mental health state was defined according to the information collected in the SF-12 items used in the health survey. The SF-12 describes a person’s health condition over a 4-week period and includes a mental health component as well as a physical component [25]. From all 12 items, a Mental Component Summary (MCS), ranging from 0 to 100, was calculated; 100 is the best status of health [25, 36]. As recommended by the User’s Manual of the SF-12v2 ® Health Survey, a norm-based cutoff point was used to distinguish between poor and good mental health [25]. The cutoff point for the self-rated mental component was set at 44.30, based on a previous study [37].

### Covariates

Sociodemographic variables and health behaviors that could influence the association between self-rated mental health and conventional medical or psychological treatment for depression were included in multivariable analyses. Age was divided into three age groups; 16 to 29 years, 30 to 59 years and 60 years and over. Ethnicity was dichotomized into ethnic Danes or immigrants and children of immigrants. Data on sex, age and ethnicity were drawn from The Central Population Register [28]. Marital status was categorized in two groups; married/cohabiting (married, registered partnership, cohabiting and cohabitants) and living alone (single) at baseline. Children that lived with their parents were coded as married/cohabiting. Data on marital status was obtained from The Danish Income Statistics Register [32]. According to United Nations Educational, Scientific and Cultural Organization’s (UNESCO) guidelines for education level classification and The International Standard Classification of Education 2011, the levels for the highest completed education at baseline were split into the following categories: basic school, high school, vocational education, short/medium education and long/high education [38]. Participants still enrolled in an education program were coded according to their highest completed education at the time of response. Education information was drawn from The Population’s Education Register [31]. Three income groups were defined based on information from The Danish Income Statistics Register; low (0–13,400 EUR pr. year), middle (13,401–26,799 EUR pr. year) and high (> 26,800 EUR pr. year) [39]. Participants were classified from DREAM [33] registrations as working or not at the time the questionnaire was answered. Smoking was dichotomized into current daily smoker or not current smoker (non-smoker, not current smoker) categories [24]. Physical activity outside work was divided into physically active (at least light exercise for a minimum of four hours a week) or not physical active categories. All health behavior information was drawn from the health survey [24].

### Statistical analyses

Data were analyzed by survey weighted cox regression analyses, and presented as hazards ratios (HR) with 95 % confidence intervals (CIs). Analyses were conducted applying design weights to correct for the different selection probabilities, and thereby, avoid biased parameter estimates. In all analyses, sex and age were included as covariates. Other preselected explanatory covariates were included in the analyses. Tests for possible interactions between age and mental health, and between sex and mental health were examined. Regardless of statistically significant interactions, the analyses were stratified into tree age groups; young (16–29 years), adults (30–59 years) and elderly (≥ 60 years) to investigate the age groups separately. The level of statistical significance was set at a *p*-value < 0.05 for all statistical analyses. For the competing risk of death, we produced crude cumulative incidence curves to examine the probability of redeeming prescriptions of antidepressants. Data management was performed using SAS version 9.4 (SAS institute Inc. Cary, North Carolina, USA). Statistical analyses were performed using R statistical software package, version 3.2.2 (R Development Core Team).

## Results

### Reporting of mental health and types of conventional medical or psychological treatment

Table 1 shows the characteristics of the study population stratified by age groups according to self-reported poor or good mental health. Poor mental health decreased across the three age groups. Among the participants aged between 16 to 29 years, 620 (22.7 %) participants reported poor mental health. In the group of participants between 30 to 59 years and the participants aged 60 years and over, 1592 (18.2 %) and 723 (15.2 %) suffered from poor mental health, respectively. During the study period, medical and psychological treatments were observed in approximately one of every five young participants. From those suffering from poor mental health, 82 (13.2 %) redeemed prescriptions of antidepressants; 27 (4.4 %) redeemed prescriptions of sedatives; and 28 (4.5 %) underwent consultation for psychological therapy. In all age groups, women were more likely than men to rate their mental health as poor, but this was most predominant among the young participants. Among the young reporting poor mental health 379 (61.1 %) were women, among the adult and the elderly reporting poor mental health 932 (58.5 %) and 358 (49.5 %) were women, respectively. In all age groups, a few participants redeemed prescriptions of sedatives and a few contacted a psychologist. Among the young reporting poor mental health, a total of 137 (22.1 %) received some form of treatment, while the number was 169 (8 %) among the young reporting good mental health. For the adult and elderly reporting poor mental health, a total of 309 (19.4 %) and 153 (21.2 %) received any form of treatment, respectively. Among the adult and elderly reporting good mental health, a total of 584 (8.2 %) and 361 (8.9 %) received some form of treatment, respectively.

**Table 1:** Characteristics of the study population

	Young		Adults		Elderly	
Mental health	Poor	Good	Poor	Good	Poor	Good
n (%)	620 (22.70)	2111 (77.30)	1592 (18.22)	7147 (81.78)	723 (15.18)	4040 (84.82)
Outcome						
Antidepressants	82 (13.23)	77 (3.65)	197 (12.37)	303 (4.24)	100 (13.83)	152 (3.76)
Sedatives	27 (4.35)	45 (2.13)	95 (5.97)	228 (3.19)	53 (7.33)	209 (5.17)
Utilization of psychologist	28 (4.52)	47 (2.23)	17 (1.07)	53 (0.74)	< 3^a^	< 3^a^
Died	< 3^a^	< 3^a^	10 (0.63)	25 (0.35)	53 (7.33)	120 (2.97)
Age (years), mean (SD)	21 (3.70)	21 (3.72)	44 (8.14)	45 (8.09)	67 (6.32)	67 (5.81)
Women	379 (61.13)	1032 (48.89)	932 (58.54)	3649 (51.06)	358 (49.52)	1867 (46.21)
Men	241 (38.87)	1079 (51.11)	660 (41.46)	3498 (48.94)	365 (50.48)	2173 (53.79)
Ethnicity						
Danish	596 (96.13)	2058 (97.49)	1512 (94.97)	6995 (97.87)	701 (96.96)	3979 (98.49)
Immigrants	24 (3.87)	53 (2.51)	80 (5.03)	152 (2.13)	22 (3.04)	61 (1.51)
Marital status						
Married/cohabiting	428 (69.03)	1580 (74.85)	1235 (77.58)	6180 (86.47)	494 (68.33)	3234 (80.05)
Living alone	192 (30.97)	531 (25.15)	357 (22.42)	967 (13.53)	229 (31.67)	806 (19.95)
Education level						
Basic school	362 (58.39)	1192 (56.47)	340 (21.36)	1217 (17.03)	349 (48.27)	1584 (39.21)
High school	132 (21.29)	435 (20.61)	56 (3.52)	254 (3.55)	8 (1.11)	35 (0.87)
Vocational education	78 (12.58)	296 (14.02)	698 (43.84)	3383 (47.33)	273 (37.76)	1576 (39.01)
Short/medium education	43 (6.94)	155 (7.34)	401 (25.19)	1834 (25.66)	83 (11.48)	695 (17.20)
Long/high education	5 (0.81)	33 (1.56)	97 (6.09)	459 (6.42)	10 (1.38)	150 (3.71)
Income						
Low	296 (47.74)	950 (45.00)	33 (2.07)	107 (1.50)	33 (4.56)	158 (3.91)
Middle	166 (26.77)	584 (27.66)	194 (12.19)	332 (4.65)	452 (62.52)	1997 (49.43)
High	158 (25.48)	577 (27.33)	1365 (85.74)	6708 (93.86)	238 (32.92)	1885 (46.66)
Occupational status						
Employed	282 (45.48)	1162 (55.05)	1151 (72.30)	6300 (88.15)	69 (9.54)	850 (21.04)
Unemployed	15 (2.42)	54 (2.56)	101 (6.34)	384 (5.37)	6 (0.83)	21 (0.52)
Student	272 (43.87)	838 (39.70)	31 (1.95)	84 (1.18)	< 3^a^	< 3^a^
Economically inactive	51 (8.23)	57 (2.70)	309 (19.41)	379 (5.30)	646 (89.35)	3165 (78.34)
Health behaviour						
Smokers	206 (33.23)	453 (21.46)	473 (29.71)	1571 (21.98)	189 (26.14)	746 (18.47)
Physically inactive	108 (17.42)	227 (10.75)	360 (22.61)	887 (12.41)	197 (27.25)	477 (11.81)

Characteristics of the participants stratified by age groups, young (16–29 years of age), adults (30–59 years of age) and elderly (≥ 60 years of age), and divided into poor and good self-reported mental health from the North Denmark Region Health Survey 2010 [24]

^a^Exact numbers not given due to regulations from Statistics Denmark [63]

Overall, the mean MCS score was 51.48 (standard deviation [SD] 8.69). The MCS score increased with age. The MCS score was 49.12 (9.18), 51.06 (8.39) and 53.61 (4.48) for young, adult and elderly participants, respectively.

### Probability for redeemed prescriptions of antidepressants

**Fig. 2:**
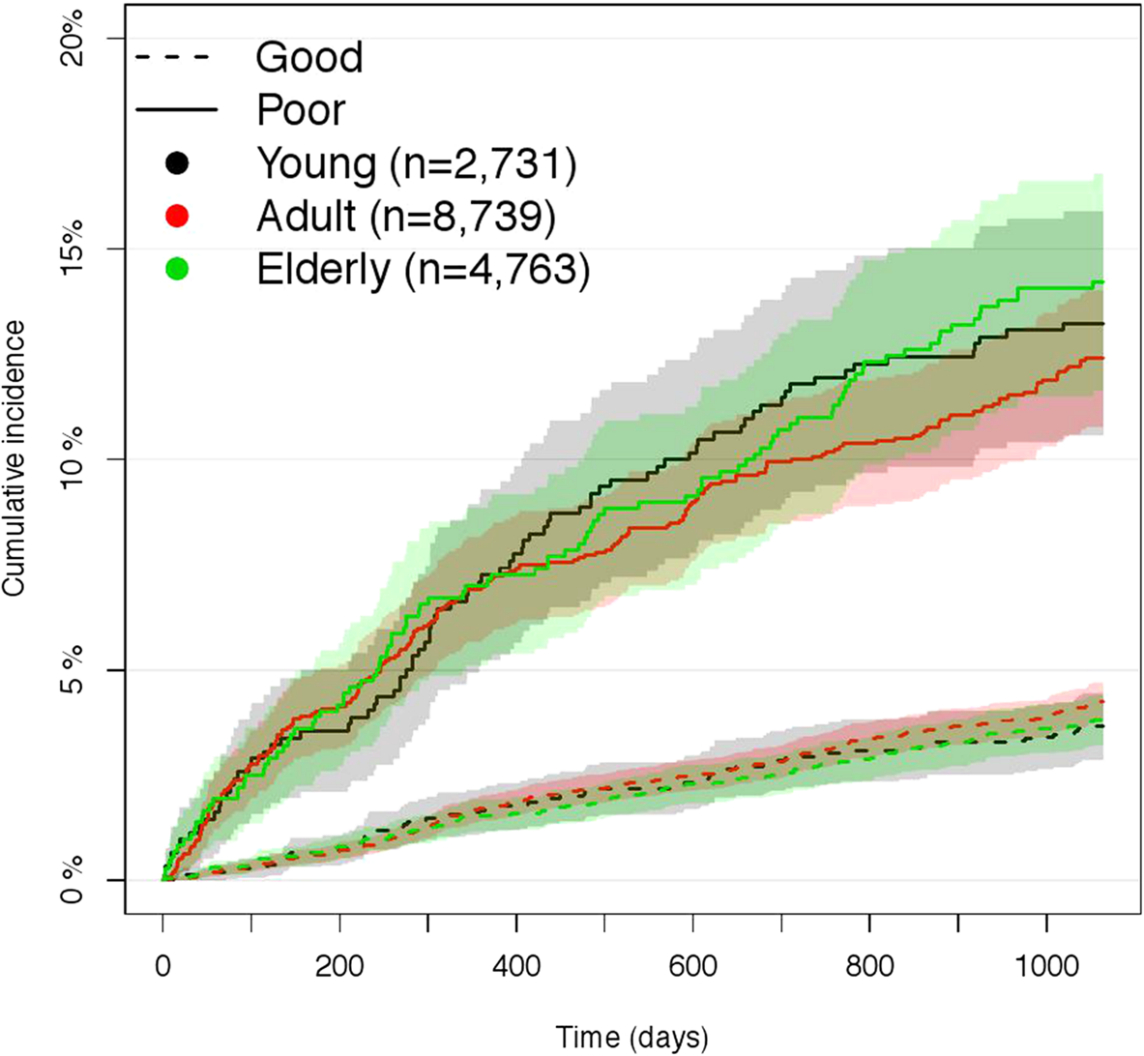
Probability for redeeming prescriptions of antidepressants. Cumulative incidence for redeeming prescriptions of antidepressants during the study period with 95 % confidence interval for poor and good mental health, stratified by age groups, young (16–29 years of age), adults (30–59 years of age) and elderly (≥ 60 years of age) from the North Denmark Region Health Survey 2010 [24]. *n* = 16,233

Cumulative incidence curves showing the unadjusted probability of redeeming prescriptions of antidepressants stratified by age groups are presented in Fig. 2. For all age groups, poor mental health was associated with an increased probability for redeeming prescriptions of antidepressants. The probability increased slightly over time for all age groups.

### The risk of redeeming prescriptions of antidepressants

**Fig. 3:**
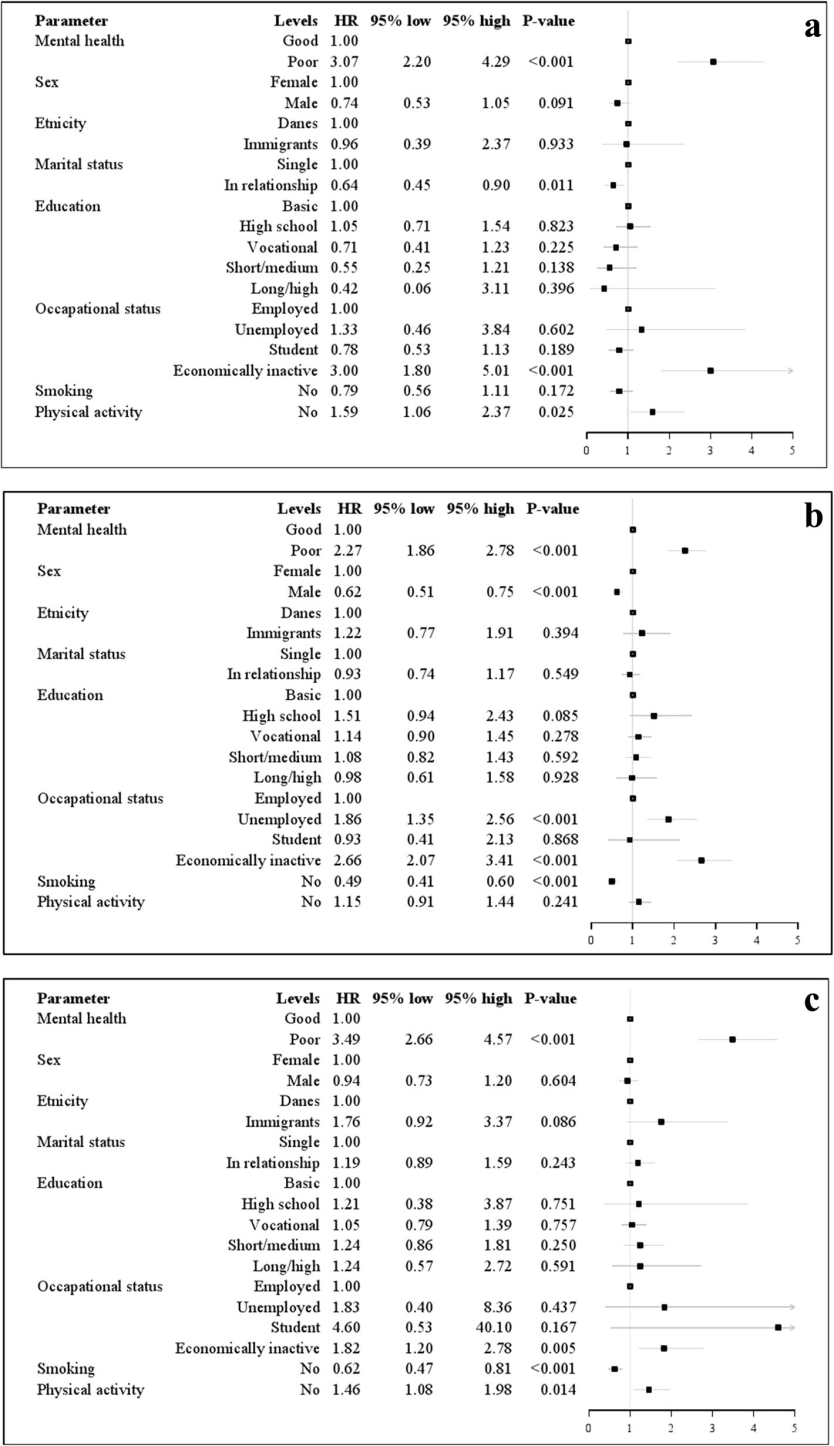
**a** Association between redeeming prescriptions of antidepressants and mental health among the young. Forest plot of hazard ratio (HR) for redeeming prescriptions of antidepressants adjusted for covariates with 95 % confidence intervals (CI) for the young (16–29 years of age) from the North Denmark Region Health Survey 2010 [24]. *n* = 2731. The unadjusted estimated HR was 3.8, 95 % CI 2.81–5.23. **b** Association between redeeming prescriptions of antidepressants and mental health among the adults. Forest plot of hazard ratio (HR) for redeeming prescriptions of antidepressants adjusted for covariates with 95 % confidence intervals (CI) for the adults (30–59 years of age) from the North Denmark Region Health Survey 2010 [24]. *n* = 8739. The unadjusted estimated HR was 3.1, 95 % CI 2.57–3.69. **c** Association between redeeming prescriptions of antidepressants and mental health among the elderly. Forest plot of hazard ratio (HR) for redeeming prescriptions of antidepressants adjusted for covariates with 95 % confidence intervals (CI) for the elderly (≥ 60 years of age) from the North Denmark Region Health Survey 2010 [24]. *n* = 4763. The unadjusted estimated HR was 4.2, 95 % CI 3.14–5.38

The survey weighted Cox regression analysis showed a statistically significant increased unadjusted HR for redeeming prescriptions of antidepressants for participants with poor mental health compared to participants with good mental health (young: HR 3.83, 95 % CI 2.81–5.23; adults: HR 3.08, 95 % CI 2.57–3.69; elderly: HR 4.11, 95 % CI 3.14–5.38). When controlling for sex, ethnicity, marital status, education level, occupational status, smoking and physical activity, the risk for redeeming prescriptions of antidepressants remained statistically significant in all age groups for participants with poor mental health compared to participants with good mental health (young: HR 3.07; 95 % CI 2.20–4.29, adults: HR 2.27; 95 %CI 1.86–2.78, elderly: HR 3.49; 95 % CI 2.66–4.57; Fig. 3a–c). Tests showed no interaction between sex and mental health. In the fully adjusted model, men had less likelihood for redeeming prescriptions of antidepressants in all age groups compared with women (young: HR 0.74, 95 % CI 0.53–1.05; adults: HR 0.62, 95 % CI 0.51–0.75; elderly: HR 0.94, 95 % CI 0.73–1.2; Fig. 3a–c). Among the young participants, the risk for redeeming prescriptions of antidepressants decreased numerically with higher education level than basic school, when controlling for the other factors, but this was not statistically significant (Fig. 3a). No interaction between mental health and educational level was found. When removing mental health from the model the risk for redeeming prescriptions of antidepressants showed same tendency among the young participants, namely a risk estimate that decreased numerically with higher education level than basic school, but this was not statistically significant. The results are presented in Additional file 1: Figure S4a-c.

### Health behaviors

In all age groups, no smoking was associated with an independent less likelihood of redeeming prescriptions of antidepressants when controlling for the other covariates. Finally, lack of physical activity was associated with a statistically significant and independent increased risk of redeeming prescriptions of antidepressants among the young and elderly participants when controlling for the other factors (young: HR 1.59, 95 % CI 1.06–2.37; elderly: HR 1.46, 95 % CI 1.08–1.98; Fig. 3a, c).

### The risk of redeeming prescriptions of sedatives and psychological therapy

Because of the small number of outcomes for redeeming prescriptions of sedatives and utilization of a psychologist, the HRs for these analyses are presented in the Additional file 2: Figure S5a–c and Additional file 3: Figure S6a, b.

## Discussion

The principal findings of this study show that self-reported poor mental health was very common among the young participants and nearly one fifth initiated treatment with antidepressants, sedatives or consulted a psychologist. Among all age groups, women were more likely to rate their mental health as poor compared with men. Poor mental health was associated with an increased probability of redeeming prescriptions of antidepressants in the 2.8-year period following the assessment of mental health in all age groups. An increased risk of redeeming prescriptions of antidepressants was observed for participants of all age groups who reported poor mental health. Among the young participants, the risk for redeeming prescriptions of antidepressants decreased numerically with higher educational levels than basic school, when controlling for all other factors, but this was a non-significant trend.

### Interpretation

In line with previous studies [13–15, 23, 40–42], our study confirms that the younger participants were more likely than the older ones to report low mental health scores. Particularly, young women are more likely than young men to report poor mental health. Larson [43] has argued that the transition from adolescence to adulthood can be stressful. It is a period in which individuals define their identity, and young women seem to be especially susceptible to feelings of insecurity and anxiety. These may influence their personal well-being and stress level [43]. Furthermore, Arnett has claimed that young women often are more explicit about concerns and fears than young men [44]. This is in line with studies showing that women more often report depressive symptoms compared to men [45–47] and thus also relevant to our study, that focuses on mental health, where depressive symptoms are a part of [4]. The suggested explanations behind the tendency that young women report poorer mental health than men, and more often than young men are strongly correlated with culture and identity factors. Understanding mental health problems among young individuals may therefore be more complex [43, 44]. As an additional explanation for the excess of poor mental health among the young participants, affective illnesses such as depression and bipolar depression, often have their onset in the age range defined in this study as the young age group. Finding effective treatment and learning to manage these conditions may reduce the proportion of participants reporting poor mental health in the two older age groups [48].

Our findings also show that of those who reported poor mental health in all age groups, the larger part did not receive conventional medical or psychological treatment. This is congruent with the findings reported in the World Health Organization (WHO) World Atlas [4].

The increased probability of redeeming prescriptions of antidepressants when reporting poor mental health for all age groups, indicates that frequent reporting of poor mental health is a common problem for all age groups and not only a phenomenon that occurs among the young. However, it should be noted that monitoring mental health in the general population has only been measured in a relatively short period of time. Nevertheless, the focus on mental health is important and requires attention, given that an association was found between poor mental health and both physical and psychiatric morbidities [49]. Conversely, many health conditions increase the risk for mental disorders and the presence of comorbidities complicates help-seeking and treatment [3].

The variation in the different age groups according to the increased risk for redeeming prescriptions of antidepressants when reporting poor mental health might be explained by different coping strategies among the different age groups [50]. Also different ways of treating mental health problem among different age groups from the health professionals may contribute to the variation [51, 52].

Our finding that risk of redeeming prescriptions of antidepressants decreased numerically with higher education levels than basic school may be interpreted as a consequence of a greater prevalence of mental health problems among people with low socioeconomic status. This findings are in line with findings from other studies showing that people with low socioeconomic status use antidepressants more often [19, 23, 53] and also report greater morbidity [54]. They are however also contrasted by two other studies that reported an increased use of antidepressants among participants with high socioeconomic status [40, 42]. This inconsistency may be explained by a difference in study samples, as these studies included only government employees, which is a relatively homogeneous group in regard to socioeconomic status [40], while our study, and others that evaluated the general population, included participants from all socioeconomic levels. It should be included in the interpretation that participants still enrolled in an education program were coded according to their highest completed education at the time of response.

### Strengths and limitations

Register data on prescriptions of antidepressants and sedatives lead to accurate follow-up, and thereby, a high validity. Additionally, inclusion of possible socioeconomic covariates drawn from registers strengthened this study, as population-based registers are not susceptible to selection bias caused by non-responders. The SF-12 instrument is recommended because of international validation [25] and the ability of SF-12 to accurately measure mental health has been documented in several studies [36, 37]. In this study, we followed the recommendations of dichotomizing mental health status as stated in the User’s Manual for the SF-12v2 ® Health Survey, which also helped strengthen this study. Nevertheless, appropriate care is needed when calculating and interpreting summary scores, such as MCS. Pelle et al. found that the SF-12 instrument carries weight when used to diagnose depression rather that anxiety, substance use, and other mental disorders [55].

The data from The Danish National Prescription Registry do not guarantee that the participants are actually using the prescribed medication. This can cause non-differential misclassification and consequent overestimation of the relation between mental health and use of antidepressants. Nevertheless, other studies have also found this analytic approach useful [6, 19, 28]. Conversely, we excluded 1379 persons from the present analysis because they had already redeemed prescriptions of antidepressants or sedatives at baseline; thus, the proportion of conventional medication and psychological treatments may be higher than in our findings. Even though the majority of antidepressants are prescribed to treat depression, it is known that antidepressants are also prescribed for other diseases [56]. In Denmark, services of both primary and specialist healthcare providers are covered by the national reimbursement system and are free of charge; however, this is not the case for the consultation and treatment by a psychologist. Patterns in medication prescription in Denmark may differ considerably from those in other countries where use of healthcare services are dependent on the individual’s financial status and health insurance coverage [23].

The standard of care in psychiatric treatment for bipolar depression does not include typical antidepressants, and the use of low doses of major sedatives and other mood stabilizers, such as lithium, is a more common approach to treating this condition [57]. However, Geddes and Miklowitz found the proportion of prescriptions of those medications in the general population to be overly small; hence, we did not evaluate these in our study [57]. It should also be mentioned that the treatment of bipolar depression with medications that do not fit the ATC code for antidepressants is possible [57].

Data obtained from the North Denmark Region Health Survey 2010 may be biased in terms of selection because of the non-response rate. Low response rates in some subgroups of the study population may affect the generalization of the study results. A previous study showed a tendency of higher response rates with the increasing education level of the subjects [58]. For these reasons, these results should be interpreted with caution, especially when extrapolating these results to the general population. A study from Finland focused on non-response in a nation-wide health study found that the main reasons for non-response might be the predisposing and socio-demographic and behavioral factors [59]. People with poor mental health may be less likely to participate in the health survey. Hence the proportions of poor mental health may be underreported. The same applies to the risk of redeeming prescriptions of antidepressants when having poor mental health. Our results may thus be underestimated for this group. The health survey was in Danish, thus excluding non-Danish speaking migrants. Migrants are known as a vulnerable group [60], which could have contributed to our study. A characteristic of non-response in the survey is found in Additional file 4: Table S2.

### Implication

This study adds information about the relationship between self-reported poor mental health and prescription of antidepressants. When reporting poor mental health, an increased probability and increased HR for redeeming prescriptions of antidepressants was observed in all age groups. Based on the above, when people report poor mental health, it should not be taken lightly. This study adds to the literature by verifying self-reported poor mental health against information in nationwide, administrative registers. Further research is needed in relation to monitored mental health over a lifespan, especially for the large group of individuals that reported poor mental health, but did not receive conventional medical or psychological treatment.

## Conclusion

This study found that self-reported poor mental health was more frequent among young participants than the older participants and that antidepressants were more often used than sedatives and consultations with a psychologist. An increased probability of redeeming antidepressant prescriptions when having poor mental health was observed in all age groups. Additionally, an increased association of self-reported poor mental health with subsequent redemption or fulfillment of antidepressant prescriptions was observed in all age groups. These findings may indicate that the frequent self-reporting of poor mental health is a common problem across all age groups that needs more attention.

## Abbreviations

ATC, Anatomical Therapeutic Chemical Classification System; CI, confidence interval; DDD, Defined Daily Dose; DREAM, Danish Register for Evaluation of Marginalization; HR, hazard ratio; MCS, Mental Component Summary; OECD, Organisation for Economic Co-operation and Development; SD, standard deviation; SF-12, Short-Form 12; UNESCO, United Nations Educational, Scientific and Cultural Organization; WHO, Word Health Organization

## Additional files

Additional file 1: Figure S4a. Redeemed prescriptions of antidepressants and mental health among the young. Figure S4b: Redeemed prescriptions of antidepressants and mental health among the adults. Figure S4c: Redeemed prescriptions of antidepressants and mental health among the elderly. (DOCX 220 kb) Additional file 2: Figure S5a. Association between redeeming prescriptions of sedatives and mental health among the young. Figure S5b: Association between redeeming prescriptions of sedatives and mental health among the adults. Figure S5c: Association between redeeming prescriptions of sedatives and mental health among the elderly. (DOCX 224 kb) Additional file 3: Figure S6a. Association between utilization of a psychologist and mental health among the young. Figure S6b: Association between utilization of a psychologist and mental health among the adult. (DOCX 157 kb) Additional file 4: Table S2. Characteristics of the non-response. (DOCX 16 kb)
