# Supplementary material for: The relationship between self-reported mental health and redeemed prescriptions of antidepressants: a register-based cohort study
Source: BMC Psychiatry. 2016 Jun 7;16:189. doi: 10.1186/s12888-016-0893-7 (PMC4897872; doi:10.1186/s12888-016-0893-7)
Supplement: Additional file 1: Figure S4a. — Redeemed prescriptions of antidepressants and mental health among the young. Figure S4b: Redeemed prescriptions of antidepressants and mental health among the adults. Figure S4c: Redeemed prescriptions of antidepressants and mental health among the elderly. (DOCX 220 kb) [file 12888_2016_893_MOESM1_ESM.docx]

# Additional file 1: Figure S4a-c

**Figure S4a**

Title: Redeemed prescriptions of antidepressants and mental health among the young

Legend: Forest plot of hazard ratio (HR) for redeeming prescriptions of antidepressants adjusted for covariates with 95% confidence intervals (CI) for the young (16–29 years of age) from the North Denmark Region Health Survey 2010 [24]. n=2,731.

**Figure S4b**

Title: Redeemed prescriptions of antidepressants and mental health among the adults

Legend: Forest plot of hazard ratio (HR) for redeeming prescriptions of antidepressants adjusted for covariates with 95% confidence intervals (CI) for the adults (30–59 years of age) from the North Denmark Region Health Survey 2010 [24]. n=8,739.

**Figure S4c**

Title: Redeemed prescriptions of antidepressants and mental health among the elderly

Legend: Forest plot of hazard ratio (HR) for redeeming prescriptions of antidepressants adjusted for covariates with 95% confidence intervals (CI) for the elderly (≥60 years of age) from the North Denmark Region Health Survey 2010 [24]. n=4,763.
