# Supplementary material for: The relationship between self-reported mental health and redeemed prescriptions of antidepressants: a register-based cohort study
Source: BMC Psychiatry. 2016 Jun 7;16:189. doi: 10.1186/s12888-016-0893-7 (PMC4897872; doi:10.1186/s12888-016-0893-7)
Supplement: Additional file 2: Figure S5a. — Association between redeeming prescriptions of sedatives and mental health among the young. Figure S5b: Association between redeeming prescriptions of sedatives and mental health among the adults. Figure S5c: Association between redeeming prescriptions of sedatives and mental health among the elderly. (DOCX 224 kb) [file 12888_2016_893_MOESM2_ESM.docx]

# Additional file 2: Figure S5a-c

**Figure S5a**

Title: Association between redeeming prescriptions of sedatives and mental health among the young

Legend: Forest plot of hazard ratio (HR) for redeeming prescriptions of sedatives adjusted for covariates with 95% confidence intervals (CI) for the young (16–29 years of age) from the North Denmark Region Health Survey 2010 [24]. n=2,731. The unadjusted estimated HR was 2.1, 95% CI 1.29–3.35.

**Figure S5b**

Title: Association between redeeming prescriptions of sedatives and mental health among the adults

Legend: Forest plot of hazard ratio (HR) for redeeming prescriptions of sedatives adjusted for covariates with 95% confidence intervals (CI) for the adults (30–59 years of age) from the North Denmark Region Health Survey 2010 [24]. n=8,739. The unadjusted estimated HR was 1.9 95% CI 1.50–2.43.

**Figure S5c**

Title: Association between redeeming prescriptions of sedatives and mental health among the elderly

Legend: Forest plot of hazard ratio (HR) for redeeming prescriptions of sedatives adjusted for covariates (sex, ethnicity, marital status, education level, smoking and physical activity) with 95% confidence intervals (CI) for the elderly (≥60 years of age) from the North Denmark Region Health Survey 2010 [24]. n=4,763. The unadjusted estimated HR was 1.5, 95% CI 1.11–2.14.
