# Supplementary material for: The relationship between self-reported mental health and redeemed prescriptions of antidepressants: a register-based cohort study
Source: BMC Psychiatry. 2016 Jun 7;16:189. doi: 10.1186/s12888-016-0893-7 (PMC4897872; doi:10.1186/s12888-016-0893-7)
Supplement: Additional file 3: Figure S6a. — Association between utilization of a psychologist and mental health among the young. Figure S6b: Association between utilization of a psychologist and mental health among the adult. (DOCX 157 kb) [file 12888_2016_893_MOESM3_ESM.docx]

# Additional file 3: Figure S6a,b

**Figure S6a**

Title: Association between utilization of a psychologist and mental health among the young

Legend: Forest plot of Hazard ratio (HR) for utilization of psychologist adjusted for covariates (sex, marital status, education level, occupational status, smoking and physical activity) with 95% confidence intervals (CI) for the young participants (16–29 years of age) from the North Denmark Region Health Survey 2010 [24]. n=2,731. The unadjusted estimated HR was 2.1, 95% CI 1.19–3.28.

**Figure S6b**

Title: Association between utilization of a psychologist and mental health among the adult

Legend: Forest plot of Hazard ratio (HR) for utilization of psychologist adjusted for covariates (sex, marital status, education level, occupational status, smoking and physical activity) with 95% confidence intervals (CI) for the adults (30-59 year old) from the North Denmark Region Health Survey 2010 [24]. n=8,739. The unadjusted estimated HR was 1.4, 95% CI 0.80–2.48.
