# Supplementary material for: The relationship between self-reported mental health and redeemed prescriptions of antidepressants: a register-based cohort study
Source: BMC Psychiatry. 2016 Jun 7;16:189. doi: 10.1186/s12888-016-0893-7 (PMC4897872; doi:10.1186/s12888-016-0893-7)
Supplement: Additional file 4: Table S2. — Characteristics of the non-response. (DOCX 16 kb) [file 12888_2016_893_MOESM4_ESM.docx]

# Additional file 4: Table S2

**Table S2**

Title: Characteristics of the non-response

|  | **Non-response** |
| --- | --- |
| n (%) | 12308 |
|  |  |
| **Outcome** |  |
| Antidepressants, missing=1166 | 970 (8.71) |
| Sedatives, missing=262 | 549 (4.56) |
| Utilization of psychologist | 100 (0.81) |
|  |  |
| Died | 875 |
|  |  |
| Age (years), mean(SD) | 49 (20.35) |
| Women | 5729 (46.55) |
| Men | 6579 (53.45) |
|  |  |
| **Ethnicity**, missing=3 |  |
| Danish | 11346 (92.21) |
| Immigrants | 959 (7.79) |
|  |  |
| **Marital status**, missing=76 |  |
| Married/cohabiting | 7297 (59.66) |
| Living alone | 4935 (40.34) |
|  |  |
| **Education level**, missing=860 |  |
| Basic school | 5320 (46.47) |
| High school | 601 (5.25) |
| Vocational education | 3880 (33.89) |
| Short/medium education | 1287 (11.24) |
| Long/high education | 360 (3.14) |
|  |  |
| **Income**, missing=69 |  |
| Low | 1685 (13.77) |
| Middle | 4059 (33.16) |
| High | 6495 (53.07) |
|  |  |
| **Occupational status**, missing=1981 |  |
| Employed | 5495 (53.21) |
| Unemployed | 431 (4.17) |
| Student | 673 (6.52) |
| Economically inactive | 3728 (36.10) |

Legend: Characteristics of the non-response from the North Denmark Region Health Survey [24] n=12,308.
